# Three intellectual disability-associated de novo mutations in *MECP2* identified by trio-WES analysis

**DOI:** 10.1186/s12881-020-01042-w

**Published:** 2020-05-11

**Authors:** Yi Gu, Bingwu Xiang, Lina Zhu, Xiuwei Ma, Xiang Chen, Tao Cai

**Affiliations:** 1 Department of Psychiatry, Beijing Children’s Hospital, Capital Medical University, National Center for Children’s Health, Beijing, 100045 China; 2 Experimental Medicine Section, National Institute of Dental and Craniofacial Research, National Institutes of Health, Bethesda, MD 20892 USA; 3 Physical Medicine and Rehabilitation Center, The Second Affiliated Hospital and Yuying Children’s Hospital, Wenzhou Medical University, Wenzhou, 325027 Zhejiang China; 4 Department of Neurology, Bayi Children’s Hospital, General Military Hospital of Beijing, Beijing, 10007 China

**Keywords:** *MECP2*, Intellectual disability (ID), Rett syndrome, Whole-exome sequencing (WES), de novo mutation (DNM)

## Abstract

**Background:**

To date, at least 746 genes have been identified to cause intellectual disability (ID). Among them, mutations in the Methyl CpG binding protein 2 (*MECP2*) gene are the leading cause of Rett syndrome and associated ID.

**Methods:**

Considering the large number of ID-associated genes, we applied trio-based whole-exome sequencing (trio-WES) and in silico analysis for genetic diagnosis of 294 children with ID.

**Results:**

Three de novo heterozygous mutations [NM_004992.3: c.502C > T, p.(Arg168*), c.916C > T, p.(Arg306Cys), and c.879C > G, p.(Ile293Met)] in *MECP2* were identified in three unrelated girls. The first two mutations were detected in two patients who were diagnosed as typical Rett syndrome, X-linked ID and psychomotor retardation. The third mutation (c.879C > G), a previously unreported, was found in a 6-year-old girl with ID, microcephaly, severe underweight and psychomotor retardation. Particularly, this extremely rare de novo mutation (DNM) is located in the transcriptional repression domain (TRD) of *MECP2*, where at least 62 different causal mutations are identified.

**Conclusions:**

We identified three DNMs in *MECP2* in a cohort of 294 individuals with ID. The novel c.879C > G mutation, as a likely pathogenic allele, may become a risk factor associated with X-linked ID, microcephaly and psychomotor retardation.

## Background

Intellectual disability (ID) involves early-onset impairments in general mental abilities [1]: 1) intellectual functioning, including reasoning, problem solving, planning, abstract thinking, judgment, academic and experiential learning; 2) adaptive functioning, including conceptual, social and practical skills. The worldwide prevalence of ID is about 10.37/1000 population [2], with an unbalanced sex ratio of 1.3–1.4 to 1 for male to females [3].

85% of patients with ID could be classified as mild ID. Environmental factors, such as malnutrition during pregnancy, perinatal asphyxia, and neurotoxic compounds exposure, are believed to play an important role in the mild ID [4]. Genetic causes are more frequently observed in affected individuals with severe ID [4]. Chromosomal abnormalities, like Down syndrome (trisomy 21) and DiGeorge syndrome (22q11.2 deletion), are the common causes of genetic ID [4]. Monogenic mutations could collectively explain a large proportion of ID cases as well [5]. At least 746 genes that influence intelligence have been identified [5]. Of note, 141 of them are located on X-chromosome [6], such as *FMR1* mutations in Fragile X syndrome and *MECP2* mutations in Rett syndrome (RTT) [7–10].

The etiology in about 50% of ID cases remains unknown [2]. Considering the large number of ID-related genes [5], the importance of genetic factors in ID [4], and genetic heterogeneity of the Rett syndrome spectrum resulting from mutations of multiple causative genes [11], we performed trio-whole-exome sequencing (trio-WES) and bioinformatics analysis to identify potential genetic causes for a cohort of 294 affected individuals with ID [11]. In the present study, we report three *MECP2*-associated de novo mutations (DNMs), including a previously unreported missense mutation.

## Methods

### Participants

294 non-homogeneous children (aged from 2 to 10 years) with intellectual disability and possible accompanying other conditions were recruited since 2010. Subjects with established perinatal diseases or chromosomal aneuploidies were not included.

### Trio-WES and bioinformatics analysis

Genomic DNAs were isolated from peripheral venous blood leukocytes. Trio-WES with 60× average sequencing depths (97% coverage) and in silico analysis were performed (Angen Gene Medicine Tech, Beijing, China) as previously described [12–14]. All mutations were identified utilizing Genome Analysis Toolkit and filtered to exclude the mutations with MAF > 0.001 in gnomAD (https://gnomad.broadinstitute.org/) or Chinese Exome Sequence Database (ChES, 5000 Chinese Han individuals, Angen Gene Medicine Tech, Beijing, China) as well as synonymous mutations that do not involve splicing. Deleterious single-nucleotide mutations were predicted by multiple commonly used algorithms, such as SIFT, PolyPhen-2, MutationTaster, and Proven programs [12–14]. Potential inherited mutations and DNMs related to neurodevelopmental disorders were screened using a recently developed program mirTrios [15].

## Results

### Mutation description

**Fig. 1:**
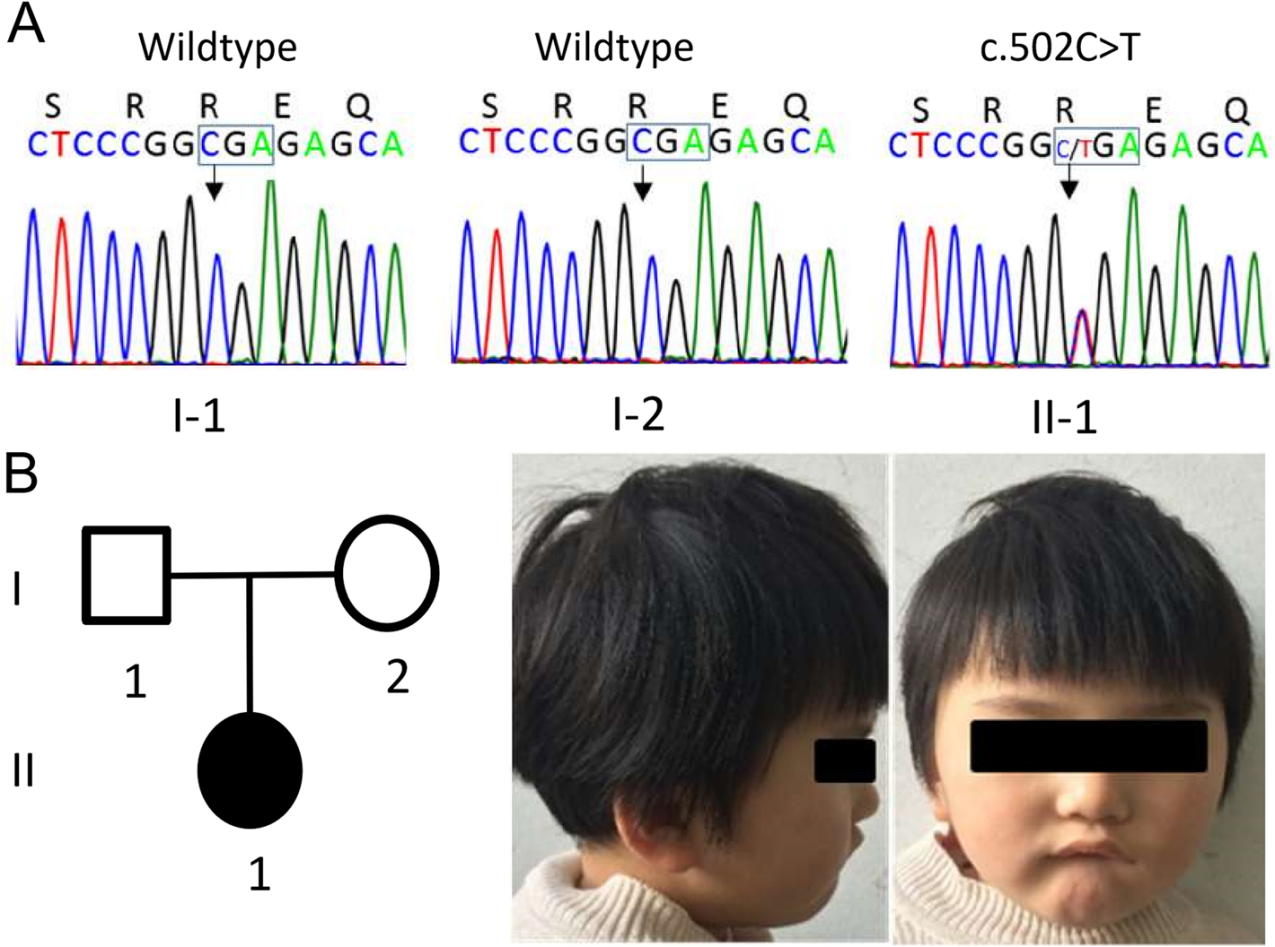
Family 1 and verification of a de novo nonsense mutation c.502C > T, p.(Arg168*) of *MECP2*. **a** The mutation is detected in the proband, but not in the parents, is confirmed by Sanger sequencing. **b** Pedigree of the family and facial photograph of the proband at 3 years old

**Fig. 2:**
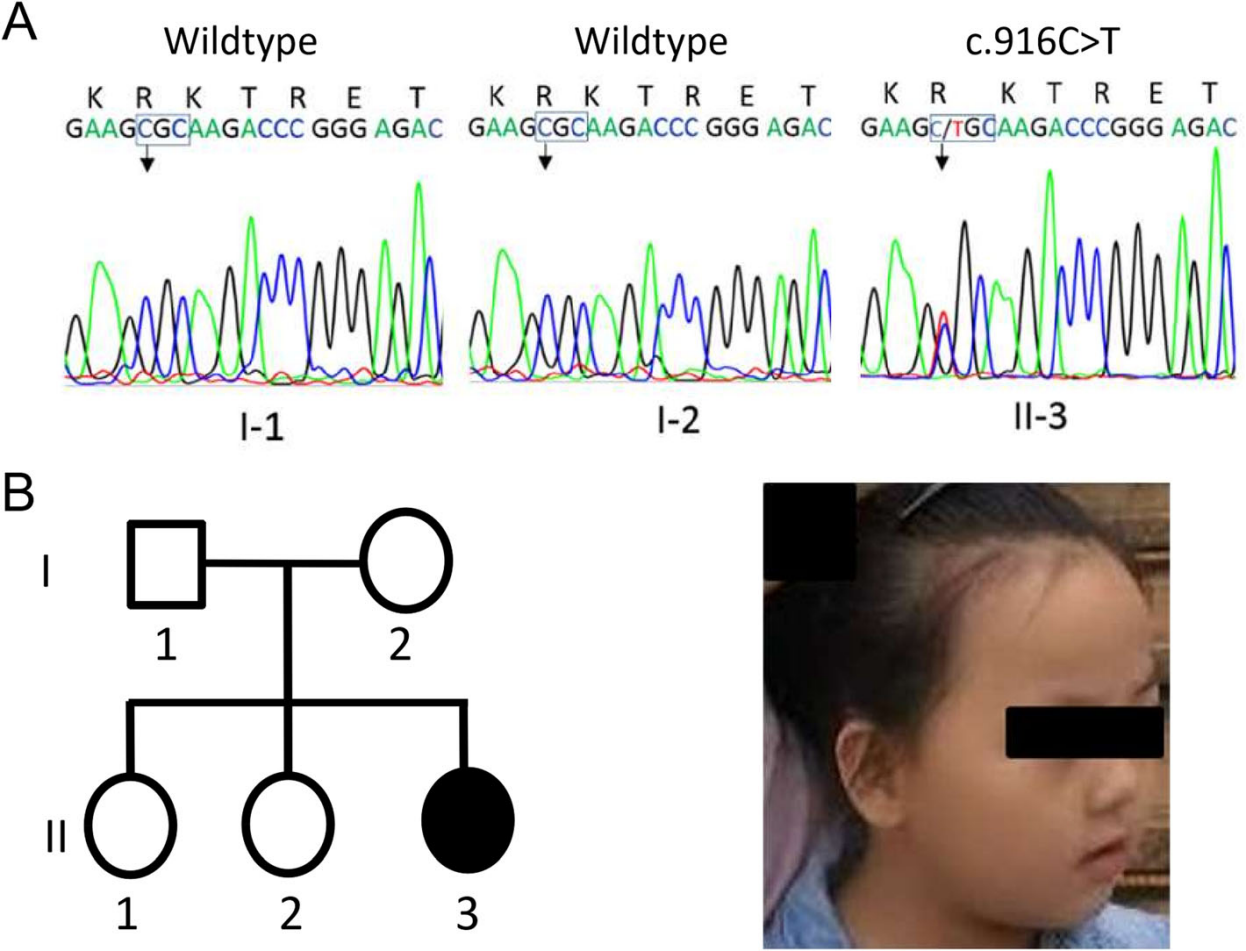
Sanger sequencing of the mutation c.916C > T, p.(Arg360Cys) of *MECP2* in case 2. **a** The mutation is present in the proband, but not in the parents, is confirmed by Sanger sequencing. **b** Pedigree of the family and facial photograph of the proband at 8 years old

A nonsense [NC_000023.10: g.153296777G > A, NM_004992.3: c.502C > T, p.(Arg168*)] and two missense mutations [NC_000023.10: g.153296363G > A, NM_004992.3: c.916C > T, p.(Arg306Cys); NC_000023.10: g.153296400G > C, NM_004992.3: c.879C > G, p.(Ile293Met)] in exon 4 of *MECP2* were identified in case 1, case 2 and case 3 respectively. All of the mutations were de novo heterozygous in the affected individuals. The c.502C > T and c.916C > T were further confirmed by Sanger Sequencing using ABI 3700 Genetic Analyzer (ABI, Foster City, CA, USA) (Fig. 1a and Fig. 2a). The c.879C > G mutation was detected in the 3rd case with high-depth reading (heterozygous, 180x vs 160×) in WES report. Sanger Sequencing was not conducted in this case because her DNA sample was no longer available.

To date, at least 1103 different mutations in *MECP2* have been curated in HGMD [16] and RettBASE [8]. Mutation spectrum analysis of the gene revealed that the first two alleles (c.502C > T, rs61748421; c.916C > T, rs28935468) we identified, like many other frequently mutated alleles [7], are located in the Methyl-CpG binding domain (MBD) and transcriptional repression domain (TBD) respectively [9].

**Fig. 3:**
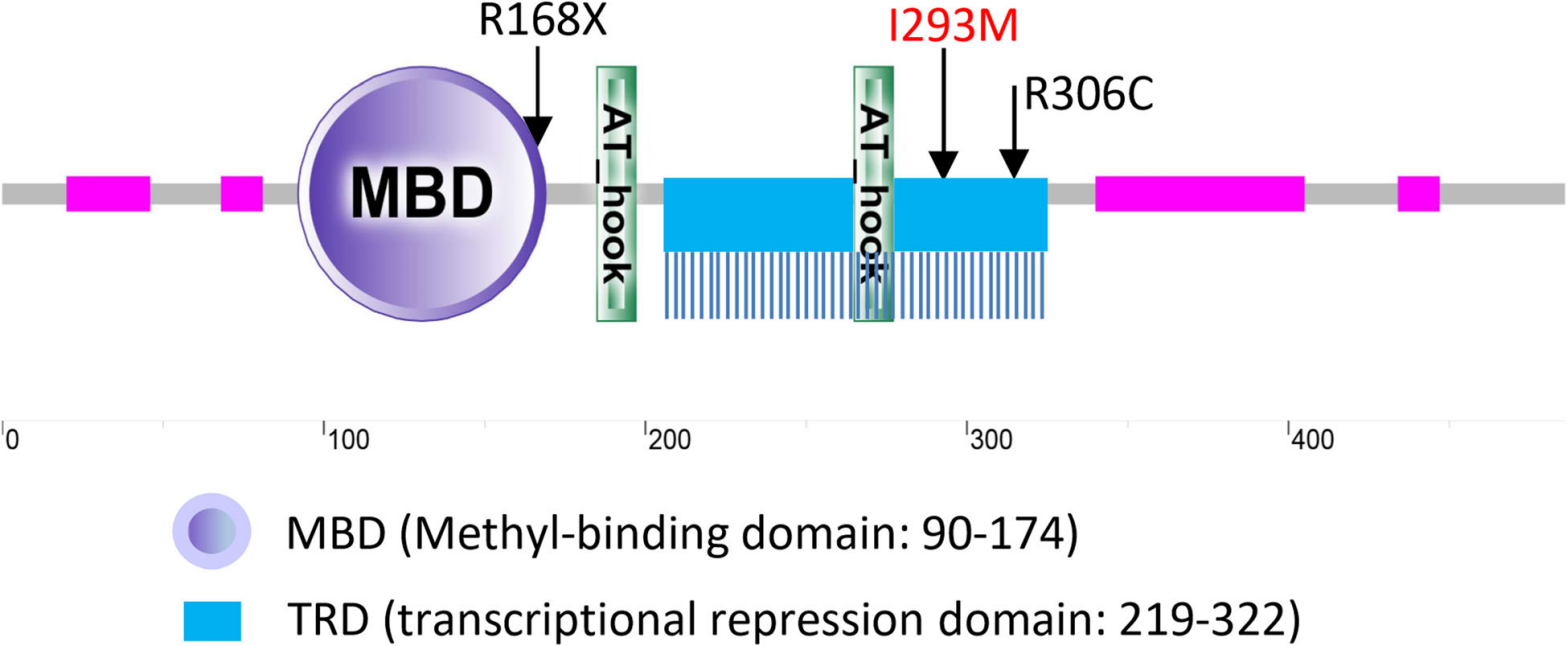
Three de novo mutations in *MECP2* identified in this study are located in MBD and TRD domain of MeCP2 protein. Location of the novel DNM p.(Ile293Met) (I293M) in TRD domain is labeled in red. Multiple vertical lines beneath the blue box represent 62 different mutations in TRD domain (HGMD), which appears to be evenly distributed within the domain composed of 104 amino acids. Both p.(Arg168*) (R168X) and p.(Arg306Cys) (R306C) mutations in the first two patients are hot alleles. MeCP2 domains are predicted by SMART analysis (http://smart.embl-heidelberg.de/). AT-hook is a DNA-binding motif. Pink boxes represent low compositional complexity regions

The novel DNM [c.879C > G, p.(Ile293Met)] dedected in the third patient is also located in TBD (Fig. 3). Within the TBD domain of 104 amino acids (from residue 219 to 322), 62 different pathogenic mutations have been identified (HGMD). In ClinVar and gnomAD, the c.879C > G allele (rs587783140) is extremely rare, which is detected in east Asian (MAF: 0.0002692) and Latino (MAF: 0.00007129), but not in any other populations. Furthmore, only 1 missense mutation p.(Thr240Ser) with a high MAF is found within the TBD domain (gnomAD), suggesting potentially benign mutations are extremely rare in this domain. Although marked as variant of uncertain significance (VUS) in ClinVar, it is predicted to be deleterious by several commonly used algorithms, such as MutationTaster, Polyphen2 and Proven. According to ACMG criteria [17], the p.(Ile293Met) missense mutation is predicted to be likely pathogenic based on the fact that it is a DNM and located in a critical and well established functional domain, rich in pathogenic variants. No additional DNMs or causal alleles, related to ID or psychomotor retardation in autosomal recessive or X-linked form, were identified from the patient in the trio-WES analysis.

### Clinical manifestations of three individuals with *MECP2* mutations

The 7-year-old girl (case 1) carrying p.(Arg168*) mutation had an uneventful perinatal period and her head circumference was 33 cm (15th, − 1 SD) at birth. She could smile and babble at 5 months, pincer grasp at 11 months, stand and walk independently between 20 and 21 months, speak “papa” and “mama” at 24 months. At 3 years and 2 months (Fig. 1b), she manifested dyspraxic gait and strephexopodia, meanwhile her purposeful hand skills started to disappear, and the repetitive involuntary hand movements arose. At 3 years and 7 months, Gesell Developmental Schedules indicated her development quotient between 11 and 19 months; auditory brainstem response threshold of Auditory Evoked Potentials (AEP) was 20 dBnHL at left and 40 dBnHL at the right side; no abnormalities were found in her brain MRI, EEG and chromosomal analysis. At 5 years and 3 months, she had bruxism and hyperventilation when awake; her head circumference was 48.5 cm (15th, − 1.5 SD), height was 104 cm (15th) and body weight was 18 kg (50th); Wechsler Intelligence Scale for Children (WISC, full-scale IQ 7) indicated profound ID. She could never crawl, climb stairs or speak phrases more than two words. Her parents were phenotypically normal (Fig. 1b).

Clinic manifestation of the patient demonstrated several features of Rett-like syndrome, such as postnatal deceleration of head growth, hand stereotypy, loss of acquired hand skills, early retardation of language and motor development. Additional conditions, such as growth retardation, breathing disturbance and bruxism when awake, were also observed in this case. Combining the genetic finding of *MECP2* pathogenic mutation, the diagnosis of typical RTT was made [10].

The 8-year-old girl (case 2, Fig. 2b), who carries the p.(Arg306Cys) mutation, was healthy at birth. She could smile at 3 months, sit steadily at 6 months, crawl and grasp at 12 months. At 24 months, she developed pincer grasp and independent stand, and responded to her name; her head circumference was 48.2 cm (50-85th). At 30 months, she began to babble, and walked with abnormal gait. At 3 years and 1 month, she was found to avoid eye-contact and interaction with others. Clancy Behavior Scale measurement suggested possible autism. Although she did not have a seizure history, EEG detected multifocal epileptic-like discharges during her sleep. At 7 years and 4 months, she showed stereotypic hand movements; her height was 117 cm (15-50th), body weight was 24 kg (50th). Brain MRI and AEP were normal. She could not run, hop, or climb stairs. She could not speak “papa” or “mama”. Due to lack of compliance, WISC and ADOS were not tested. Her family members were phenotypically normal.

Although this patient had hand stereotypy, her normal head growth did not support the diagnosis of RTT [10]. Considering her overall developmental delay and autistic-like features after 6 months, the diagnosis of X-linked ID, psychomotor retardation and possible Autism Spectrum Disorder (ASD) was made [18]. It is estimated that 16.7–40% of ID patients have ASD, whereas ID is seen in 50–70% of ASD patients [19]. Sometimes ID, ASD, RTT and other neurologic diseases share similar clinical features, which has been a challenge in clinical diagnosis.

The 6.5-year-old girl (case 3), who was found to carry p.(Ile293Met) mutation, demonstrated motor retardation in a few months after birth. Her head circumference of 45 cm (< − 3 SD) and weight of 11.2 kg (< − 3 SD). Anterior fontanelle was closed. No abnormalities were found in examination of her heart and lung. Her walking is slower compared to the kids at similar age, probably due to reduced lower-extremity muscle strength (4/5 grade). However, examinations of bilateral knee and ankle tendon reflexes, bilateral Babinski sign, and bilateral ankle clonus were normal. At 3-year-old, her Psychomotor Development Index (PDI) score in Bayley Scales test was only 50 (< 69 for developmental delay), while her Mental Development Index (MDI) score was 70 (< 70 for developmental delay). Her sleep EEG was abnormal, showing few sharp waves and sharp slow waves on both front sides of brain. But no seizure history was documented. Cranial MRI showed abnormal signals in white matter. She was diagnosed as psychomotor retardation, ID, microcephaly and severe underweight. No further clinical information was available because she was lost to follow-up. Her parents were phenotypically normal.

## Discussion

MeCP2 protein is abundantly expressed in brain tissues [9], and selectively binds to DNA sequences with methylated CpG dinucleotides, interacting with histone deacetylases and the transcriptional co-repressors [7, 9]. Extensive target genes have been discovered in recent studies, suggesting that MeCP2 is a genome wide epigenetic modulator [9]. Accordingly, *MECP2* mutations have been demonstrated to cause various clinical conditions (HGMD), such as Rett syndrome (678 different mutations), *MECP2* duplication syndrome (85), X-linked ID (52), neurodevelopmental delay (35), brain abnormalities (26), atypical Rett syndrome (22), and psychomotor developmental delay (14). It has been observed that a majority of patients with *MECP2*-caused RTT are sporadic [7, 20] because most pathogenic mutations in *MECP2* are de novo mutations [21]. In many cases, DNMs are likely derived from the X chromosome in male sperms [22, 23].

In terms of genotype-phenotype correlations, previous studies showed that females carrying p.Arg168* in MBD domain could manifest more severe symptoms compared to p.(Arg306Cys) [7] in TRD domain (Fig. 3). However, case 2 with p.(Arg306Cys) in our study showed more severe language impairment and extra autistic features compared to case 1 with p.(Arg168*). As shown in Fig. 3, the novel DNM p.(Ile293Met) in case 3 is also located in the TRD domain. Notably, a total of 62 different mutations (HGMD) have been found in this domain that is composed of only 104 amino acids, suggesting its important function to regulate the methylated CpG sites on the DNA strands involved in brain development. Therefore, it is likely that this allele, as a DNM present in the affected case 3, may play a role in the pathogenesis of the her clinic conditions.

Wide phenotypic variations in our cases could be also observed in many other individuals with the same *MECP2* mutations. Part of the reasons may be attributed to the existence of many other factors, such as X chromosome inactivation, somatic mosaicism and genetic background [7, 9, 24]. Further genomic sequencing and in-depth bioinformatic analysis may identify more risk mutations in affected individuals with broader neurodevelopmental diseases, which may allow a better deciphering of the pathophysiology of neurodevelopmental disorders.

## Conclusions

Three DNMs in the *MECP2* gene, including the previously unreported missense mutation p.(Ile293Met), are identified by trio-WES analysis from 294 children with ID. Our findings may expand the *MECP2*-associated mutational and phenotypic spectrum in affected individuals with ID and other accompanying conditions.

## Data Availability

The datasets generated and analysed during the current study are available [doi:10.17632/34v7c9rkgf.1] in the Mendeley Data repository (10.17632/34v7c9rkgf.1). No additional supporting files are available for further studies.

## Abbreviations

MECP2: Methyl CpG binding protein 2
ID: Intellectual disability
WES: Whole-exome sequencing
DNM: de novo mutation
RTT: Rett syndrome
DNA: Deoxyribonucleic acid
MAF: Minor allele frequency
HGMD: Human Gene Mutation Database
MBD: Methyl-CpG binding domain
TBD: Transcriptional repression domain
VUS: Variant of uncertain significance
ACMG: The American College of Medical Genetics and Genomics
SD: standard deviation
ADOS: Autism Diagnostic Observation Schedule
AEP: Auditory Evoked Potentials
MRI: Magnetic resonance imaging
EEG: Electroencephalo-graph
WISC: Wechsler Intelligence Scale for Children
IQ: Intelligence quotient
ASD: Autism spectrum disorder
PDI: Psychomotor Development Index
MDI: Mental Development Index
